# A study of the transit amplification divisions during spermatogenesis in *Oncopetus fasciatus* to assess plasticity in sperm numbers or sperm viability under different diets

**DOI:** 10.1002/ece3.4511

**Published:** 2018-10-03

**Authors:** Ashley E. Duxbury, Brandie Weathersby, Zachary Sanchez, Patricia J. Moore

**Affiliations:** ^1^ Department of Entomology University of Georgia Athens Georgia

**Keywords:** cell cycle, cost of reproduction, life‐history trade‐off, nutrition, *Oncopeltus fasciatus*, sperm quality, spermatogenesis

## Abstract

*Oncopeltus fasciatus* males fed the ancestral diet of milkweed seeds prioritize reproduction over lifespan as evidenced by higher rates of fertility and shorter lifespans than males from the same population fed the adapted diet of sunflower seeds. We examined the proximate mechanisms by which milkweed‐fed males maintained late‐life fertility. We tested the hypothesis that older milkweed‐fed males maintained fertility by producing more, higher quality sperm. Our results, that older males have more sperm, but their sperm do not have higher viability, are in general agreement with other recent studies on how nutrition affects male fertility in insects. We further examined the mechanisms by which sperm are produced by examining the progression of spermatogonial cells through the cell cycle during the transit amplification divisions. We demonstrated that diet affects the likelihood of a spermatocyst being in the S‐phase or M‐phase of the cell cycle. Given work in model systems, these results have implications for subtle effects on sperm quality either through replication stress or epigenetic markers. Thus, viability may not be the best marker for sperm quality and more work is called for on the mechanisms by which the germline and the production of sperm mediate the cost of reproduction.

## 1 INTRODUCTION

A fundamental tenant of life‐history theory is that there is a trade‐off between reproduction and lifespan (Stearns, 1992). However, the mechanisms by which the cost of reproduction is manifest have been elusive (Flatt, 2011; Harshman & Zera, 2007; Speakman, 2008). Few studies have identified the mechanisms underlying phenotypic plasticity in the trade‐off under different environmental conditions, even though we expect individuals to have the ability to strategically adjust expenditure on gametes given the likelihood that a particular reproductive opportunity will result in fitness benefits. The best‐studied example is dietary restriction, defined as reduced food without malnutrition. Dietary restriction leads to an increase in lifespan, usually with a reduction in reproductive rates, and we assume that this plasticity is adaptive, allowing organisms to maximize fitness to specific environmental conditions (e.g., Zajitschek et al., 2016).

Much of the work on the physiology of the reproduction–lifespan trade‐off has focused on females and the cost of producing expensive gametes (Hayward & Gillooly, 2011), yet males also experience significant costs of reproduction. Research on reproductive costs for males has focused on mate searching, courtship, and male–male competition under different environments, both social and nutritional environments (Flatt & Heyland, 2011; Hunt et al., 2004; Scharf, Peter, & Martin, 2013; Shuker & Simmons, 2014). It is now clear, however, that the sperm production represents a significant cost to males and researchers are exploring phenotypic plasticity in sperm numbers and quality under variable social and nutritional environments (Bunning et al., 2015; Dávila & Aron, 2017; Joseph, Sasson, Allen, Somjee, & Miller, 2016; Moatt, Dytham, & Thom, 2014). *Drosophila melanogaster* males exposed to the odor of a rival male store both more sperm and a greater proportion of live sperm in their seminal vesicles (Moatt et al., 2014). High‐quality nutrition, on the other hand, appears to promote increased sperm numbers, but does not impact sperm quality in cockroaches (Bunning et al., 2015), leaf‐footed bugs (Joseph et al., 2016), or ants (Dávila & Aron, 2017).

While all of these studies document an outcome of environmental variation on sperm quantity and quality, none examined the mechanisms by which the increase in sperm numbers occurred. Ultimately, sperm availability depends on the germline, cells set aside for the production of gametes (Extavour, 2013; Moore, 2014). Males have the potential, through germline stem cells, to modulate sperm production (Kaczmarczyk & Kopp, 2011; Moore, 2014; Ramm & Schärer, 2014). While we have many studies examining the developmental and genetic controls on germline stem cells, these cells are rarely examined in an evolutionary context. Further, the energetic cost of producing gametes may not represent the full, or even major, cost of reproduction (Maklakov & Immler, 2016). Maintaining genomic and proteomic integrity within the germline may be more costly in males than females, given the increased rate of turnover in the germline stem cells required for producing numerous sperm.

**Figure 1:**
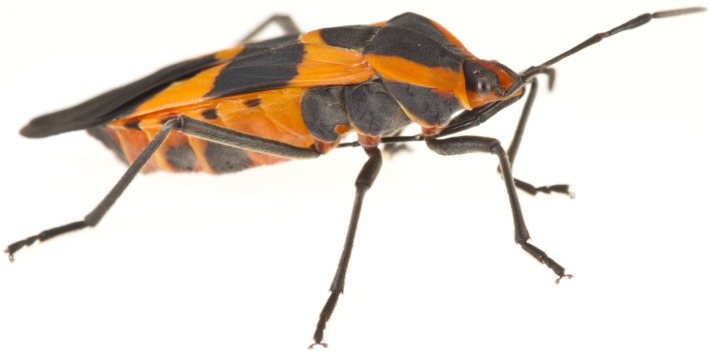
The milkweed bug, *Oncopeltus fasciatus*. The milkweed bug has been used as a model organism in ecology and evolution and in evolutionary developmental biology. Photograph courtesy of Jena Johnson Photography (http://jenajohnson.zenfolio.com/about.html)

For this study, we took advantage of an experimental laboratory population of the milkweed bug, *Oncopeltus fasciatus* (Figure 1). While the usual host plant for *O. fasciatus* in the wild is members of the family Asclepiadaceae (Feir 1974), the commercially available laboratory population has been reared exclusively on sunflower seeds for over 400 generations. The laboratory population of *O. fasciatus* is able to use both the ancestral food of milkweed seeds and the adapted diet of sunflower seeds, demonstrating a diet expansion rather than evolving specialization during adaptation to the sunflower seed diet (Newcombe, et al., 2015; Newcombe, Moore, & Moore, 2015). Females from this population show no difference in fitness on the two diets (Moore & Attisano, 2011). Males, however, while having equal lifetime reproductive success, demonstrate different patterns of life‐history trade‐offs on the two diets (Attisano, et al., 2012). Milkweed‐fed males prioritize reproduction over lifespan by maintaining late‐life fertility. Males on the milkweed diet mate more frequently and fertilize more eggs than males on the sunflower diet, but these differences only become significant when the males are over a month past adult eclosion. Milkweed‐fed males pay a cost to the increase in reproductive investment. Milkweed‐fed males live a maximum of 60 day postadult eclosion while sunflower‐fed males live to 90 days and lifespan is significantly affected by diet (Attisano et al., 2012).

**Figure 2:**
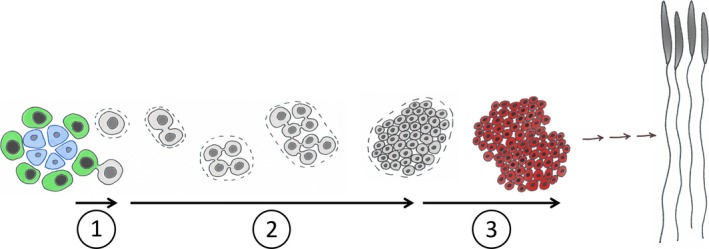
In *Oncopeltus fasciatus*, the germline stem cell niche is a rosette of cells at the tip of each testis tubule (blue cells; Schmidt et al., 2004). Germline stem cells (GSC; green cells) are in contact with the niche, which is essential to maintaining GSC identity. Spermatogenesis is initiated by a GSC dividing to produce one daughter cell that remains in the niche and remains a stem cell and another daughter cell that moves away from the niche and becomes a spermatogonial cell (Step 1; grey cells). Spermatogonial cells are encapsulated by cyst cells (dashed line) and undergo a series of mitotic transit divisions to form a 64 cell spermatocyst (Step 2; Ewen‐Campen et al., 2013). The diploid spermatogonial cells then undergo meiosis to form the haploid spermatocytes (Step 3; red cells) that will differentiate into mature spermatids

Here, we tested the hypothesis that reduced late‐life fertility in sunflower‐fed males was due to reduced sperm production, reduced sperm quality, or a combination of reduced sperm numbers and sperm quality. We also examined the developmental mechanism which could give rise to any potential change in sperm numbers. Spermatogenesis requires a series of events, any one of which could be affected by diet (Figure 2). Variation in the rate of germline stem cell division to produce spermatogonial cells, the rate at which the spermatogonial cells undergo transit amplification divisions to form spermatocysts, or the rate of entry into meiosis to produce spermatocytes will result in variation in the rate of sperm production (Ramm & Schärer, 2014). We predicted that older milkweed‐fed males would have a higher sperm viability than sunflower‐fed males. We also predicted that older milkweed‐fed males would have more sperm stored in their seminal vesicles due to an increase in the rate of transit amplification divisions.

## 2 METHODS

### 2.1 Animal husbandry

All colonies and individuals were kept in incubators at 26°C and 16:8 L:D. Eggs were collected from stock colonies and left to mature through 5th instar in a nymphal colony with sunflower seeds and water. On the day of adult emergence, experimental males were put into individual dishes with either organic, unsalted sunflower seeds (FoodtoLive.com; sunflower‐fed), or milkweed seeds (Everwilde.com; milkweed‐fed) and water. Newly emerged females were put into colonies with sunflower seeds and water and kept to provide males with virgin females. While we did not measure adult size in either males or females, individuals were randomly assigned to treatment groups. Thus, any potential effect of size on fertility or fecundity was distributed across treatment groups.

Males were randomly assigned to either the milkweed seed or sunflower seed diets. Twenty males were assigned to each food treatment. Experimental males were mated at 2 weeks, 3 week, and 4 week postadult eclosion. The 2‐week mating was to assess fertility at a young age, the 4‐week mating was to assess fertility at an old age. The 3‐week mating was to maintain the experimental conditions used in the Attisano et al. (2012) study but we did not assess fertility or fecundity in these females in this experiment. Mating trials were carried out as described in Attisano et al. (2012). The food was removed from the male's dish to prevent any effect of food treatment on female fecundity. A female was then introduced to the dish. All pairs were observed until the first mating. Pairs were placed back into the incubators and were allowed to mate over a period of 48 hr.

### 2.2 Fertility and fecundity

For the 2‐ and 4‐week mating trials in which male fertility was being assessed, we controlled for effect of female age by using females that were 7–10 day postadult eclosion and were virgins at the time of the mating trials. The females for the 3‐week mating trials were of unknown age and mating status, but all mated within the first few hours of the mating trial. These females were returned to the mass colony following the mating trial and not used in the fecundity and fertility data. The virgin females from the 2‐weed and 4‐week mating trials were placed in individual petri dishes with water, sunflower seeds, and cotton wool for laying eggs after their 48‐hr mating with the experimental male. *Oncopeltus fasciatus* eggs are pale yellow when they are laid and develop a deep red color as the embryo develops, allowing embryo development to be scored visually. Eggs were collected every 3 days and left in the incubator to mature until they reached a red color, usually 5 days, indicating they were fertilized and had initiated development. Eggs were then counted and scored as fertilized or not fertilized based on their color. The females were left to lay eggs for the rest of their lives, which lasted about 4 to 5 weeks, and egg numbers combined to examine lifetime fecundity (total eggs produced) and fertility (percent of eggs fertilized) for mates of sunflower‐ and milkweed‐fed males.

### 2.3 Quantity and quality of sperm

To determine whether diet affects either the quantity or quality of sperm produced by males, we assessed both the numbers of sperm stored in the sperm storage organ, the seminal vesicle, and viability of stored sperm of 4 week posteclosion males on each diet. Twenty males were randomly assigned to each diet treatment. Males in this experiment had only mated once and then given 10 days to recover sperm stores prior to dissection. Males were dissected into PBS, and one seminal vesicle was used for sperm quantity and the other for sperm quality assays.

Sperm quantity was determined by counting the number of sperm from the seminal vesicle (Montrose, Harris, Moore, & Moore, 2008). The seminal vesicle was placed into 200 ultrapure water and gently ground with a micropestle. A 4 μl aliquot of the sperm solution was diluted into 600 μl ultrapure water, and 10 μl of 0.5% EosinY was added to the dilution to improve contrast on the slide. The diluted sperm was then placed onto slides in a series of ten 10 μl spots. The spots were allowed to dry, and all sperm in the 10 spots were counted. The total number of sperm from the seminal vesicle was calculated.

Sperm quality was assayed using the LIVE/DEAD Sperm Viability kit (Thermo Fisher Scientific) using the method of Montrose et al. (2008). This kit uses a cell membrane permeable green fluorescent stain (SYBR 14) and the membrane impermeable stain propidium iodide to differentiate between living and dead cells. Living cells are labeled green due to the presence of the SYBR 14 and the exclusion of the propidium iodide. Dead or dying cells are unable to exclude the propidium iodide and so are stained red. The second seminal vesicle was placed in 1 ml testis isolation buffer (TIB; 183 mM KCl, 47 mM NaCl, 10 mM Tris, pH 6.9: Parrott, Hudson, Brady, & Schulz, 2012) +10% bovine serum albumin and gently broken open with a micropestle. 100 μl of the sperm solution was placed into 900 μl of TIB and SYBR 14 and propidium iodide added to the sperm solution. After 10 min at room temperature, 12 samples from each seminal vesicle were placed on a welled slide and imaged with an AMG EVOS FL microscope (Thermo Fisher Scientific, USA). Slides were imaged with both the GFP (showing the SYBR 14 staining) and the RFP (showing the propidium iodide staining) EVOS LED light cubes (Thermo Fisher Scientific, USA) and one image containing at least 10 sperm cells was taken of each of the 12 wells. Images were randomized and counted blind with respect to treatment. The number of living (green) and dead/dying (red) cell were counted for each male. It was apparent early in the experiment that the sperm viability is always very high regardless of treatment (mean of 98% viable) and that there was little variation (*SE* = 0.7%), so data were only collected on a subset of samples (seven from each diet treatment) as it was time intensive and not informative.

### 2.4 Germline staining

Thirty males were randomly assigned to each food treatment. Males were chosen randomly to be dissected at young age (one day after the 2‐week mating trial), or old age (one day after the 4‐week mating trial). Testes were removed from the male and placed into TIB. Individual testioles were separated and removed from the surrounding testis membrane prior to fixation. Testioles were assayed ex vivo for cell proliferation using two different markers of the cell cycle. The cell cycle consists of a series of stages. Cells must first replicate their DNA during the S‐ (for synthesis) phase. The duplicated DNA is then divided into two daughter cells during the M‐ (for mitotic) phase during which the replicated chromosomes are separated. Finally, the separated chromosomes and cytoplasm of the mother cell are divided into two separate cells during cytokinesis. We assayed two of these three stages, the S‐phase and M‐phase. First, we assayed for cells in the S‐phase using the ClickiT EdU Alexa Fluor 647 imaging kit (Thermo Fisher Scientific C10340). EdU is incorporated into DNA during the S‐phase of the cell cycle and is then visualized with a fluorescent tag. We also stained testioles for cells in the M‐phase of the cell cycle using a polyclonal antibody against Histone H3 phosphorylated at serine 10 (pHH3), a modification specific to the mitotic phase of the cell cycle (Millipore Sigma Antibody 06‐570). Labeling two phases of the cell cycle allows researchers to distinguish between variation in length of the entire cell cycle (in which the change observed should be consistent among the two markers) or variation in a single phase of the cell cycle (in which the results with two markers will be discordant.)

Individual testioles were incubated in TIB and EdU for 45 min at RT to allow for incorporation of EdU into cells in the S‐phase of the cell cycle. After EdU incorporation, testioles were fixed in 4.5% formaldehyde in phosphate saline buffer (PBS) for 30 min at RT. Following washes in PBS plus 0.1% Tween‐20 (PBT) and 5% normal goat serum, the EdU was labeled with the 647 (cyan) fluorescent ClickiT reagent for 30 min in the dark at RT. Testioles were then incubated with the primary pHH3 antibody, followed by a goat anti‐rabbit secondary antibody labeled with an Alexa Fluor 488 fluorescent (green) marker.

**Figure 3:**
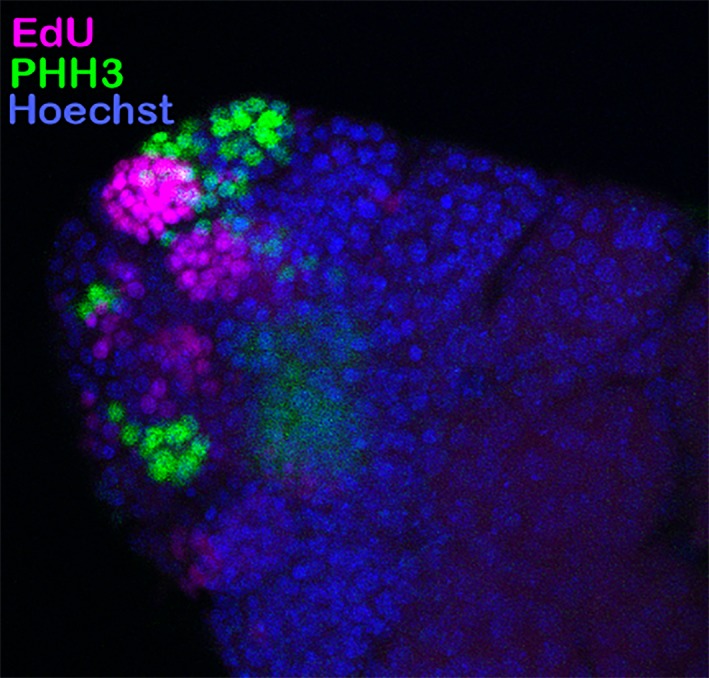
Example micrograph for transit amplification cell division cycles. This photograph represents a typical staining pattern within the testiole showing spermatocysts stained for various stages of the cell cycle. Testis tubules are stained for S‐phase through the incorporation of EdU during DNA replication. The same testis tubules are also stained for the M‐phase, with the antiphosphohistone H3 (PHH3) antibody against a mitosis‐specific histone modification. It is apparent that all spermatogonial cells within the spermatocyst are synchronized as all nuclei within the spermatocyst are only labeled with a single cell cycle marker if they are stained at all. For details, see the Section 2

The stained testes were then imaged using a Zeiss LSM 710 Confocal Microscope (Zeiss) at the UGA Biomedical Microscopy Core and an EVOS Fl Cell Imaging system (Thermo Fisher). Three testioles from each male were imaged and analyzed. Images were coded and counted blind by two independent operators. For each testiole, the number of spermatocysts stained positive for anti‐pHH3 antibody or EdU was counted and the total for all three testioles from each male added together (see Figure 3).

### 2.5 Statistical analyses

Statistical analyses were done using JMP Pro v 13.0.0. For the fertility and fecundity experiments, male reproductive success was assessed across two ages. Therefore, for this experiment, we used a repeated‐measures ANOVA. The experiment on sperm numbers and sperm viability, only a sperm from the older males was examined. These data were analyzed with a one‐way ANOVA using diet as the factor. Analysis of the cell cycle was carried out on both 2‐week‐ and 4‐week‐old males, but due to the destructive sampling required each age represents a different group of males. Therefore, the data were analyzed with a two‐way ANOVA using diet and age as factors.

## 3 RESULTS

### 3.1 Fertility and fecundity of females based on male diet

**Figure 4:**
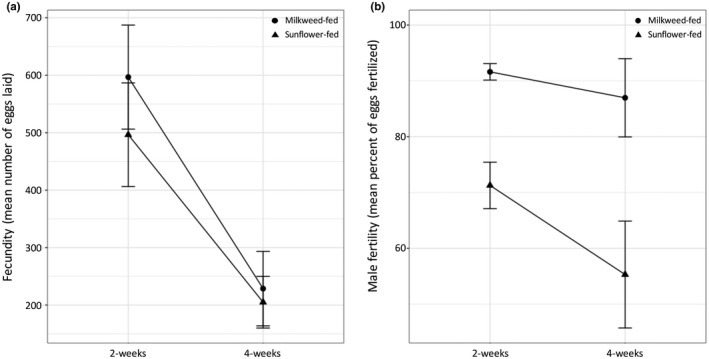
Male fertility and fecundity depend on age and diet. (a) The mean numbers of eggs laid by 7‐ to 10‐day‐old females mated to males early in life is greater than the number of eggs laid by 7‐ to 10‐day‐old females mated to the same male later in life. (b) Milkweed‐fed males fertilized a higher proportion of eggs laid by their mates than sunflower‐fed males at both ages. Error bars represent *SE*

In this experiment, the fecundity of females mated to young (2‐week‐old) males was statistically significantly higher than those mated to old (4‐week‐old) males (repeated‐measures ANOVA within‐subjects *F*_1,27_ = 15.35, *p* < 0.001); Figure 4a). However, there was no statistically significant difference in fecundity of females due to the diet of their mate and no interaction between age and diet (repeated‐measures ANOVA between‐subjects *F*_1,27_ = 0.93, *p* = 0.343; diet*age *F*_1,27_ = 0.21, *p* = 0.652). Age did not statistically significantly affect the fertility of males on either diet (repeated‐measures ANOVA within‐subjects *F*_1,27_ = 3.30, *p* = 0.80; Figure 4b), but diet did have a statistically significant effect on fertility (repeated‐measures ANOVA between‐subjects *F*_1,27_ = 13.48, *p* = 0.001). Milkweed‐fed males had higher fertility than sunflower‐fed males at both ages, and there was no interaction between age and diet (diet*age *F*_1,27_ = 0.10, *p* = 0.327).

### 3.2 Quality and quantity of sperm

**Figure 5:**
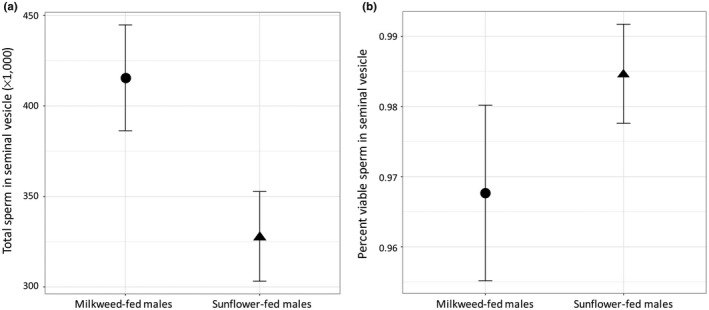
Diet affected sperm numbers, but not sperm viability. (a) Milkweed‐fed males had more sperm in their seminal vesicles at 4 week postadult eclosion than sunflower‐fed males at the same age. (b) Males on both diets maintained high sperm viability, and there was no difference with diet. Error bars represent *SE*

Old milkweed‐fed males had statistically significantly greater numbers of sperm stored in their seminal vesicles than old sunflower‐fed males (*F*_1,38_ = 5.225, *p* = 0.028; Figure 5a). Diet did not effect of the viability of the sperm within the seminal vesicle, however (*F*_1,12_ = 1.400, *p* = 0.260; Figure 5b). The viability of sperm isolated from the seminal vesicle from both milkweed‐fed and sunflower‐fed males was close to 100%.

### 3.3 Germline division rates

**Figure 6:**
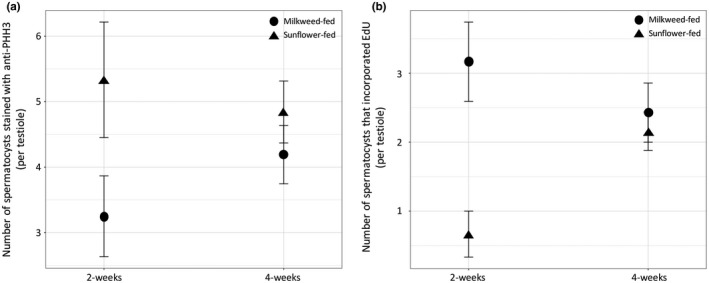
Diet, but not age, affects the progression of spermatogonial cells through the transit amplification division cell cycle. (a) Sunflower‐fed males have more spermatocysts that stain for the M‐phase of the cell cycle. (b) Milkweed‐fed males have more spermatocysts that stain for the S‐phase of the cell cycle, although the difference disappears over time. Error bars represent *SE*

The effect of diet and age on the phase of the cell cycle of spermatogonial cells within the testis tubules was complex. There was no statistically significant effect of either diet or age, and no statistically significant interaction between diet and age, for the total number of spermatocysts that are dividing and thus stain positive for either S‐phase or M‐phase (EdU and anti‐pHH3 stained spermatocysts combined: diet, *F*_1,54_ = 0.00, *p* = 0.981; age, *F*_1,54_ = 0.62, *p* = 0.433; diet*age, *F*_1,54_ = 0.27, *p* = 0.603). There was a statistically significant effect of diet on total numbers of spermatocysts staining positive with the mitosis stage‐specific marker, anti‐pHH3 antibody (*F*_1,54_ = 4.94, *p* = 0.030). Overall, sunflower‐fed males have more spermatocysts that stain positive for mitosis than milkweed‐fed males (Figure 6a). There was no statistically significant effect of age (*F*_1,54_ = 0.13, *p* = 0.716) and no statistically significant interaction between diet and age (*F*_1,54_ = 1.35, *p* = 0.250). For the marker of the DNA synthesis (S‐) phase of the cell cycle, EdU incorporation, there was a statistically significant effect of diet (*F*_1,54_ = 7.86, *p* = 0.007; Figure 6b). There was no statistically significant effect of age (*F*_1,54_ = 0.58, *p* = 0.450), but there was a statistically significant interaction between age and diet (*F*_1,54_ = 5.09, *p* = 0.028). Interestingly, milkweed‐fed males are much more likely to have spermatocysts that stain positive for S‐phase within young males, although that difference goes away with age (Figure 6b).

If the rate of sperm production under the two diets varied due to a simple speeding up or slowing down of the cell cycle proportionally, the results using the markers for S‐phase and M‐phase should be the same for the treatments, which was not the case. Thus, the relationship between the two different stages of the cell cycle was investigated further by testing for a correlation between the two different stains. In the 2‐week‐old males, there is a statistically significant negative correlation between the total number of spermatocysts staining positive for Edu and anti‐pHH3 (*r* = −0.601, *p* = 0.008), indicating that spermatocysts at this age are dividing in relative synchrony and are more likely to be observed in the M‐phase than the S‐phase of the cell cycle. This correlation disappears in the 4‐week‐old males (*r* = 0.099, *p* = 0.542).

## 4 DISCUSSION

In laboratory, populations of the milkweed bug *Oncopeltus fasciatus* males fed the ancestral diet of milkweed seed prioritize reproduction over lifespan both behaviorally and physiologically (Attisano et al., 2012). Older milkweed‐fed males mate more often and fertilize more eggs than sunflower‐fed eggs. Here, we examined the mechanism responsible for maintaining fertility later in life in these males. We asked whether older milkweed‐fed males maintain sperm production as compared to males fed of sunflower seeds. Additionally, given the increasing evidence that sperm quality declines with age, we asked whether or not older milkweed‐fed males maintain their fertility through increased sperm quality. Our results support the hypothesis that older milkweed‐fed males maintain late‐life fertility by maintaining sperm numbers. However, older milkweed‐fed males did not have higher sperm quality, measured as viability, than sunflower‐fed males. In fact, sperm samples from all males were always close to fully viable and showed almost no variability. These results are in general agreement with other recent studies on how impacts male fertility in insects. Dietary restriction has been shown to lead to a trade‐off between sperm viability and immune function in crickets (Simmons, 2012); however, this study did not examine sperm numbers. Several recent studies have shown that poor‐quality food reduces sperm numbers, but not sperm viability in cockroaches (Bunning et al., 2015) and Argentine ants (Dávila & Aron, 2017), and reduces testes mass, but not sperm viability in leaf‐footed cactus bugs (Joseph et al., 2016). Thus, a reduction in male fertility under variable food environments is often a consequence of reduced sperm production in insects.

Although our experimental design was slightly different, our results generally agreed with Attisano et al. (2012). As in Attisano et al. (2012), females mated to older males had lower fecundity. And milkweed‐fed males had higher fertility than sunflower‐fed males. The difference in fertility is documented in Attisano et al. (2012) and here can be explained by an increase in the numbers of sperm stored in the seminal vesicle of older milkweed‐fed males compared with older sunflower‐fed males. Males that have more sperm stored will have more sperm available to inseminate their mates. While there are a growing number of studies documenting the effect of nutrition on sperm quantity and quality in insects (see citations in Bunning et al., 2015), fewer have examined the consequences of this variation on male fitness. In this study, we observed a positive correlation between male fitness (measured as percent of eggs fertilized) and sperm production. Thus, as was recently observed in the cockroach *Nauphoeta cinerea* (Bunning et al., 2015) and the flour beetle *Tribolium castaneum* (Fedina & Lewis, 2006), sperm production appears to directly translate into fitness. However, that is not the case in all systems. An increase in sperm production on a high‐quality diet in *D. melanogaster* did not result in higher paternity (McGraw et al., 2007), although this study was the only one of these to examine larval as opposed to adult nutritional environment. Adult diet may be particularly important in insects where spermatogenesis proceeds throughout adulthood (Joseph et al., 2016).

Male fertility is affected by sperm quality as well as numbers. It is becoming increasingly clear that sperm quality can decline with age and environmental condition males’ experience (Marshall, 2015; Pizzari, Dean, Pacey, Moore, & Bonsall, 2008). One way that milkweed‐fed males may maintain late‐life fertility is through maintaining sperm quality. Therefore, we tested for changes in sperm viability among the older males fed the two diets. Sperm viability was uniformly high across the diets. This corresponds to what we might predict for species that are likely to experience high levels of sperm competition (Hunter & Birkhead, 2002), such as *O. fasciatus,* which mates promiscuously. Thus, the higher fertility in males fed the ancestral diet of milkweed seeds over those fed the adapted diet of sunflower seeds appears at the surface to be mediated through maintaining sperm production rather than maintaining sperm quality. However, as we discuss below, sperm viability may not be the best way of measuring sperm quality.

Clearly sperm production is costly, and as such diet will have an impact on sperm production and fertility (e.g., Bunning et al., 2015). Sperm production is influenced by energy acquisition. Bunning et al. (2015) show that in the cockroach, *N. cinerea* sperm production, but not sperm viability, increased with increased intake of nutrients. The same response was documented in male ants (*Linepithema humile*), where decreasing protein intake results in decreasing sperm numbers without a change in sperm viability (Dávila & Aron, 2017). But how does this affect the trade‐off between reproduction and lifespan? Variation in reproductive effort, such as variation in sperm production, does not inevitably result in a change in lifespan, and yet this negative correlation between fertility and lifespan is ubiquitous. Survival costs of reproduction could arise through competitive allocation of a limited resource pool, direct costs via damage to the soma such as accumulation of reactive oxygen species, or antagonistic signaling between the germline and the soma (Aguilaniu, 2015; Edward & Chapman, 2011; Flatt, 2011; Kaczmarczyk & Kopp, 2011; Maklakov & Immler, 2016). Alternatively, nutritional environment could cause a change in the physiological state of the organism that has independent, but opposite, effects on reproduction and lifespan (Aguilaniu, 2015).

Milkweed‐fed males maintain late‐life fertility but pay a cost by reduced lifespan (Attisano et al., 2012). Milkweed‐fed males are not simply better fed and thus able to invest in more sperm. Rather, the ancestral diet of milkweed is altering the life‐history trade‐off between reproduction and lifespan. It has been argued that to better understand the nature of this trade‐off, we need to understand the proximate mechanisms underlying it (Aguilaniu, 2015; Flatt, 2011; Hansen, Flatt, & Aguilaniu, 2013; Harshman & Zera, 2007). We examined the developmental mechanisms of sperm production under the two diets. Our results were not clear cut. While the number of sperm stored within the seminal vesicles was significantly different, there was no difference in the numbers of spermatocysts undergoing transit amplification divisions by either age or diet. Diet did, however, influence the stage of the cell cycle that we were likely to detect in the testioles of males. Thus, the spermatocysts in the testes of milkweed‐fed males were more likely to be in the S‐phase of the cell cycle than sunflower‐fed males, while spermatocysts of sunflower‐fed males were more likely to be in the M‐phase. And while we did not find an overall effect of age on transit amplification divisions, we found that younger males tended to have spermatocysts dividing synchronously, while that synchrony breaks down in the older males.

Our result on transit amplification divisions within the testioles is not easily reconciled with our phenotypic results on sperm numbers. We think there may be several reasons that could account for this. First, because these males were not mating frequently, the sperm stored in the seminal vesicles could have come from spermatogenesis occurring across the males’ lifetime. Thus, our sperm counts on older males would reflect spermatogenesis that occurred at younger ages. Second, while the rate of transit amplification divisions is one avenue to produce variation in sperm numbers, other steps in the process may also impact sperm production (Figure 2). While the germline stem cells and stem cell niche have been identified morphologically in *O. fasciatus* (Schmidt & Dorn, 2004; Schmidt, Papanikolaou, & Dorn, 2001), we do not currently have the molecular markers to identify the male germline stem cells or the stem cell niche, and thus, we are unable to measure variation in the rate at which spermatogonial cells are born (Figure 2, step 1) as has been done in *Drosophila melanogaster*. In *D. melanogaster*, the numbers of germline stem cells in the testes vary with both age (Wang & Jones, 2011) and diet (McLeod, Wang, Wong, & Jones, 2010; Wang, McLeod, & Jones, 2011). Thus, stem cells can respond directly to the nutritional status and thus represent a potential avenue for coordinating diet and fertility (Kaczmarczyk & Kopp, 2011; Moore, 2014). We are working to identify cell markers such that we can directly assess germline stem cell dynamics rather than using the indirect measure of transit amplification division rates. Variation in the rate at which spermatocysts transition to meiosis also could affect sperm numbers (Figure 2, step 3). *Deleted in Azoospermia* is a highly conserved gene family involved in male fertility (VanGompel & Xu, 2011). The ancestral gene in the family, *boule*, is found in invertebrates, including *O. faciatus* (Ewen‐Campen, Jones, & Extavour, 2013), and a threshold level of Boule protein is required for the progression of spermatogonia into meiotic divisions (VanGompel & Xu, 2011). We have preliminary evidence that *boule* expression is upregulated in the testes of sunflower‐fed males (AED & PJM, unpublished data). If the threshold of Boule protein is reached in the spermatogonia of sunflower‐fed males earlier, it would result in spermatogonia dividing meiotically to form spermatocytes after fewer transit amplification divisions and thus result in fewer sperm cells. Further work on how testes dynamics under variable nutrient environments is needed to determine exactly how diet impacts sperm numbers in these males.

While it was unclear how milkweed‐fed males produce higher sperm numbers from our data on the cell cycle, the observation that milkweed‐fed males have spermatogonia that spend more time in the S‐phase of the cell cycle is of interest in terms of sperm quality. It has recently been proposed that the cost of the cellular mechanisms for quality control and repair required to maintain the germline integrity may represent a hidden cost of reproduction (Maklakov & Immler, 2016). The variation in progression through transit amplification divisions may result because one diet, sunflower seeds, induces a physiological state that prioritizes somatic maintenance over germline integrity and could reduce fitness not by reducing gamete production but by reducing gamete quality (but not viability) and thus offspring viability. The mechanism by which environment and age may affect sperm quality is unclear, but emerging evidence in humans indicates that tissue‐specific changes in epigenetics may influence sperm quality. Genomewide analysis has documented hypermethylation of DNA in poor‐quality sperm, and the epigenome is affected by both age and nutrition (Sharma et al., 2015). In vitro fertilization is more likely to result in a successful pregnancy if methylation in sperm is low, although there is no change in fertilization rates. If milkweed‐fed males maintained late‐life fertility simply by maintaining the cell cycle and the rate of transit amplification divisions, we would expect both of the cell cycle markers to be increased in milkweed‐fed males compared with sunflower‐fed males. The lack of agreement in the diet‐dependent changes in the S‐phase and M‐phase of the cell cycle in the spermatocysts indicates that there is a change in progression through the cell cycle. One potential explanation for fewer spermatogonia in the S‐phase in sunflower‐fed males is that the transit amplification divisions in spermatogonia are delayed at the S‐phase checkpoint in these males. Replication stress, which can be caused by nutritional limitations, will activate the S‐phase checkpoint (Mirkin & Mirkin, 2007). As the replication fork stalls, the unwound DNA is vulnerable to damage. Another possibility is that the spermatogonia of milkweed‐fed males spend more time synthesizing their DNA, and perhaps this improves the efficacy or fidelity of the replication of epigenetic marks (Kheir & Lund, 2010). Both of these mechanisms could result in the sperm of milkweed‐fed males being of higher quality, measured as the ability to support offspring development as opposed to sperm viability.

## 5 CONCLUSION

*Oncopeltus fasciatus* males show phenotypic plasticity in the reproduction–lifespan trade‐off under variable nutritional environments (Attisano et al., 2012). Given that genetic and developmental tools exist for *O. fasciatus*, this system represents an opportunity to examine the proximate mechanism underlying this central life‐history trade‐off. Here, we have examined the developmental progression of spermatocysts to explore how variation in nutritional environment might result in variation in sperm numbers, and ultimately fitness of the males. What we have demonstrated is that the pathway from diet to sperm production is not simple. Males do not simply speed up the assembly line. The results from the cell cycle markers, along with increased understanding of how sperm quality can vary with age and environment, lead us to speculate that sperm viability may not be the best measure of sperm quality leading to male fitness. However, *O. fasciatus* provides an additional model for which we can use a molecular toolkit to untangle proximate mechanisms underlying the cost of reproduction.

## AUTHOR CONTRIBUTIONS

The project was conceived and designed jointly by AED and PJM. AED and PJM jointly analyzed the data. AED wrote the original draft of the manuscript with assistance from PJM. BW collected and contributed to the analysis of the data on fecundity and fertility (Figure 4) and ZS collected and contributed to the analysis of the data on seminal vesicle sperm counts and sperm viability (Figure 5). Both BW and ZS worked in the laboratory for research credit as a part of their undergraduate degrees.

## DATA ACCESSIBILITY

Data available from the Dryad Digital Repository: https://doi.org/10.5061/dryad.q57n9h7.
